# Beyond HPV in Eastern Europe: Genotype Distribution, Molecular Biomarkers, Vaginal Microbiome, and Implications for Cervical Cancer Prevention

**DOI:** 10.3390/life16061039

**Published:** 2026-06-22

**Authors:** Eugenia-Alina Radu, Corina-Ioana Anton, Cristian-Sorin Sima, Adrian Streinu-Cercel

**Affiliations:** 1 County Clinical Emergency Hospital “Sf. Ap. Andrei”, 900591 Constanta, Romania; eugenia-alina.radu@rez.umfcd.ro; 2 Faculty of General Medicine, Carol Davila University of Medicine and Pharmacy, Bulevardul Eroii Sanitari 8, 050474 Bucharest, Romania; adrian.streinucercel@umfcd.ro; 3 Department of Medico-Surgical and Prophylactic Disciplines, Titu Maiorescu University, 040441 Bucharest, Romania; 4 Department of Infectious Diseases, “Dr. Carol Davila” Central Military Emergency University Hospital, Calea Plevnei 134, 010242 Bucharest, Romania; 5 Department of Infectious Diseases I, Faculty of Medicine, Carol Davila University of Medicine and Pharmacy, 020021 Bucharest, Romania; 6 National Institute for Infectious Diseases “Prof. Dr. Matei Balş”, Strada Doctor Calistrat Grozovici 1, 021105 Bucharest, Romania

**Keywords:** HPV, cervical cancer screening, HPV genotyping, CINtec PLUS, HPV E6/E7 mRNA, vaginal microbiome, Eastern Europe, molecular biomarkers, HPV vaccination, cervical intraepithelial neoplasia

## Abstract

Human papillomavirus (HPV) infection remains the principal etiological factor in cervical cancer development worldwide, with Eastern Europe continuing to demonstrate disproportionately high cervical cancer incidence and mortality rates. Regional disparities in screening implementation, vaccination coverage, and HPV genotype distribution contribute substantially to the persistent burden of HPV-related disease. In recent years, increasing attention has focused on molecular biomarkers and the vaginal microbiome as complementary approaches for improving cervical cancer prevention strategies. This systematic review aimed to evaluate recent evidence regarding HPV genotype distribution, molecular biomarkers, vaginal microbiome composition, and their implications for cervical cancer prevention in Eastern Europe. A systematic literature search was conducted in PubMed/MEDLINE, Scopus, Web of Science, Embase, and the Cochrane Library for studies published between January 2020 and May 2026. This systematic review was conducted in accordance with the PRISMA 2020 guidelines and prospectively registered in PROSPERO (CRD420261391136). Studies from Eastern European populations reporting data on HPV genotype distribution, screening strategies, vaccination, molecular biomarkers, or vaginal microbiome composition were included. HPV prevalence in screening populations ranged from approximately 12% to over 20%, with HPV16 consistently identified as the predominant genotype across all included studies. However, non-16/18 high-risk genotypes, particularly HPV31, HPV51, HPV52, HPV66, and HPV68, represented a substantial proportion of infections in several Eastern European cohorts. Studies evaluating CINtec PLUS cytology and HPV E6/E7 mRNA testing demonstrated improved specificity for identifying clinically significant cervical lesions compared with HPV DNA testing alone. Emerging evidence also suggested associations between vaginal dysbiosis, increased microbial diversity, persistent high-risk HPV infection, and progression to cervical intraepithelial neoplasia. Although the 9-valent HPV vaccine provides coverage for most circulating high-risk genotypes identified in the region, vaccination uptake remains inconsistent throughout Eastern Europe. The findings of this systematic review support the growing importance of extended HPV genotyping, molecular biomarkers, and microbiome-related approaches in cervical cancer prevention strategies in Eastern Europe. Strengthening organized screening programs, expanding vaccination coverage, and improving access to molecular diagnostic technologies remain essential priorities for reducing the regional burden of HPV-related disease.

## 1. Introduction

Human papillomavirus (HPV) infection remains the most common sexually transmitted viral infection worldwide and represents the principal etiological factor in the development of cervical cancer [1,2]. Persistent infection with high-risk HPV genotypes, particularly HPV16 and HPV18, is strongly associated with cervical carcinogenesis and progression to invasive cervical cancer [1,2,3]. Although the global burden of cervical cancer has declined in countries with organized screening programs and widespread HPV vaccination, cervical cancer incidence and mortality remain disproportionately elevated in several Eastern European countries [4,5,6]. Limited implementation of organized HPV-based screening, unequal access to preventive healthcare services, and inconsistent vaccination uptake continue to contribute substantially to the regional burden of HPV-related disease [4,6].

Recent epidemiological studies have demonstrated considerable heterogeneity in HPV genotype distribution across Eastern Europe [6,7,8]. While HPV16 consistently remains the predominant oncogenic genotype, several investigations have reported substantial prevalence of non-16/18 high-risk HPV genotypes, including HPV31, HPV51, HPV52, HPV66, and HPV68, in women with persistent infection, abnormal cytology, and high-grade cervical lesions [4,8,9]. In certain Eastern European cohorts, these genotypes approached or exceeded the prevalence of HPV18, suggesting a broader genotype diversity than that traditionally reported in Western European populations [7,9]. Such findings may have important implications for cervical cancer prevention strategies, particularly regarding extended HPV genotyping, molecular triage approaches, and implementation of broader-spectrum prophylactic vaccines [10,11].

In parallel with advances in HPV genotyping, increasing attention has focused on molecular biomarkers capable of improving risk stratification among high-risk HPV-positive women [11,12]. Although HPV DNA testing demonstrates high sensitivity for detecting women at risk for CIN2+ lesions, its relatively low specificity may result in increased referral rates and unnecessary colposcopy procedures, particularly in younger populations with transient HPV infections [11,13]. Consequently, biomarker-based triage strategies have emerged as important complementary approaches in modern cervical cancer screening algorithms. Among these, CINtec PLUS cytology, based on simultaneous detection of p16 and Ki-67 expression within the same cervical epithelial cell, has demonstrated promising clinical utility for identifying transforming HPV infections associated with high-grade lesions [13,14]. Similarly, HPV E6/E7 mRNA testing provides information regarding transcriptionally active infection and viral oncogene expression, potentially offering greater specificity for clinically significant disease compared with HPV DNA testing alone [12,15].

Another emerging area of interest in cervical carcinogenesis research is the role of the vaginal microbiome in HPV persistence and progression to cervical neoplasia [15,16]. *Lactobacillus*-dominant vaginal microbiota are generally associated with cervical health, lower inflammatory status, and increased viral clearance, whereas dysbiotic microbiota characterized by increased microbial diversity and anaerobic bacterial predominance have been associated with persistent high-risk HPV infection and progression to CIN2/CIN3 lesions [16,17]. Several studies have suggested that vaginal dysbiosis may contribute to epithelial barrier disruption, altered mucosal immunity, chronic inflammation, and increased oncogenic viral activity [17]. Nevertheless, microbiome-related evidence in Eastern European populations remains relatively limited and fragmented.

Despite growing interest in molecular diagnostics and microbiome-related mechanisms in cervical cancer prevention, substantial disparities remain across Eastern Europe regarding screening accessibility, vaccination implementation, and adoption of molecular diagnostic technologies [5,6]. In addition, evidence regarding HPV genotype diversity, biomarker utilization, and microbiome composition within Eastern European populations has not yet been comprehensively synthesized. Therefore, the aim of this systematic review was to evaluate recent evidence regarding HPV genotype distribution, molecular biomarkers, vaginal microbiome composition, and their implications for cervical cancer prevention strategies in Eastern Europe.

## 2. Materials and Methods

### 2.1. Review Design

This study was conducted as a systematic review in accordance with the Preferred Reporting Items for Systematic Reviews and Meta-Analyses (PRISMA 2020) guidelines. The review aimed to evaluate recent evidence regarding HPV genotype distribution, molecular biomarkers, vaginal microbiome composition, and their implications for cervical cancer prevention in Eastern European populations. This systematic review was conducted in accordance with the PRISMA 2020 guidelines and prospectively registered in the International Prospective Register of Systematic Reviews (PROSPERO; CRD420261391136).

### 2.2. Search Strategy

A comprehensive literature search was conducted in PubMed/MEDLINE, Scopus, Web of Science, Embase, and the Cochrane Library for studies published between 1 January 2020 and 1 May 2026. Additional manual searches of reference lists and Google Scholar were performed to identify potentially relevant studies not captured through database searching.

The search strategy combined both the regional term ‘Eastern Europe’ and the names of individual countries expected to contribute relevant literature. Country-specific terms were included to maximize sensitivity because studies are frequently indexed using national rather than regional descriptors. The selected countries were based on commonly used geopolitical classifications of Eastern Europe and countries with documented cervical cancer burden and available HPV-related research. Nevertheless, studies from other countries could have been underrepresented if they were not indexed using the selected search terms.

Boolean operators (“AND”, “OR”) were applied to combine search terms appropriately.
The PubMed search strategy included the following terms:
((“human papillomavirus” OR HPV)
AND
(“genotype distribution” OR genotyping OR “high-risk HPV”)
AND
(“Eastern Europe” OR Romania OR Poland OR Croatia OR Latvia OR Serbia OR Bulgaria OR Hungary OR Slovakia OR Lithuania)
AND
(screening OR vaccination OR “p16/Ki-67” OR CINtec OR “HPV mRNA” OR “E6/E7 mRNA” OR microbiome OR microbiota)).
Equivalent adapted search strategies were applied for Scopus, Web of Science, Embase, and the Cochrane Library.

### 2.3. Eligibility Criteria

Studies were considered eligible if they fulfilled the following inclusion criteria:

Inclusion criteria:

- Published between January 2020 and May 2026.
- Conducted in Eastern European populations or included clinically relevant Eastern European subgroup data.
- Reported data regarding HPV prevalence, HPV genotype distribution, cervical cancer screening, HPV vaccination, molecular biomarkers, including CINtec PLUS cytology or HPV E6/E7 mRNA testing, or vaginal microbiome composition.
- Original peer-reviewed observational or interventional studies.
- Published in English.

Exclusion criteria:

- Case reports.
- Editorials, commentaries, conference abstracts, or letters without full-text availability.
- Animal or in vitro studies.
- Studies lacking genotype-specific, biomarker-related, or clinically relevant outcome data.
- Duplicate publications.

### 2.4. Study Selection

All records identified through database searching were imported into a reference management software program, and duplicate studies were removed prior to screening. Two independent reviewers screened titles and abstracts according to predefined inclusion and exclusion criteria. Full-text articles considered potentially eligible were independently assessed for final inclusion.

Discrepancies regarding study eligibility were resolved through discussion and consensus between reviewers.

### 2.5. Data Extraction

Data extraction was performed independently using a standardized data extraction form developed for this review. The following variables were extracted from each eligible study:

- Country of origin.
- Publication year.
- Study design.
- Study population and sample size.
- HPV prevalence.
- Most prevalent HPV genotypes.
- Presence of multiple HPV infections.
- HPV vaccination data.
- Screening methodology.
- CINtec PLUS or p16/Ki-67 findings.
- HPV E6/E7 mRNA findings.
- Vaginal microbiome characteristics.
- Main clinical outcomes.

Extracted data were reviewed for consistency and accuracy prior to qualitative synthesis.

### 2.6. Quality Assessment

Methodological quality and risk of bias were assessed using the Newcastle–Ottawa Scale (NOS) adapted for cross-sectional and cohort studies. Studies scoring 7–9 points were considered high quality, 4–6 moderate quality, and ≤ 3 low quality. The assessment evaluated study selection, comparability of study populations, outcome assessment, methodological consistency, and potential sources of bias. Quality assessment was performed independently by two reviewers, with disagreements resolved through consensus. The results are summarized in Table 1.

### 2.7. Data Synthesis

Due to substantial heterogeneity among included studies regarding study populations, laboratory methodologies, reported outcomes, screening strategies, and molecular diagnostic approaches, quantitative meta-analysis was not considered appropriate. Therefore, findings were synthesized using a qualitative narrative approach.

Thematic synthesis was performed according to the following predefined categories:

1. HPV prevalence and genotype distribution.
2. Molecular biomarkers and screening implications.
3. HPV vaccination and public health impact.
4. Vaginal microbiome and HPV persistence.
5. Risk stratification and future cervical cancer prevention strategies.

Findings were subsequently compared across countries and study populations to identify regional trends, emerging patterns, and clinically relevant implications for cervical cancer prevention in Eastern Europe.

## 3. Results

### 3.1. Study Selection

**Figure 1:**
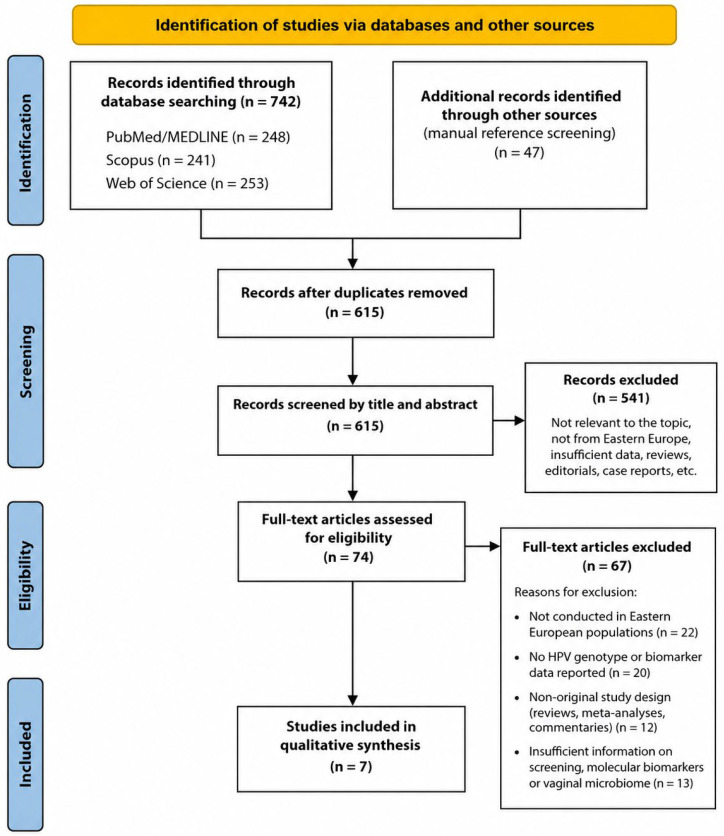
PRISMA 2020 flow diagram for study selection.

The study selection process is summarized in the PRISMA 2020 flow diagram (Figure 1). A total of 742 records were identified through database searching in PubMed/MEDLINE, Scopus, Web of Science, Embase, and the Cochrane Library. An additional 47 records were identified through manual searches of reference lists and Google Scholar.

After removal of duplicate records, 615 studies remained for title and abstract screening. Following initial screening, 74 full-text articles were assessed for eligibility. Of these, 7 studies specifically fulfilled the predefined inclusion criteria for inclusion in the final qualitative synthesis focused on Eastern European populations and molecular HPV-related outcomes.

The principal reasons for exclusion were:

- lack of relevance to Eastern European populations;
- absence of HPV genotype-specific data;
- insufficient information regarding molecular biomarkers or vaginal microbiome composition;
- non-original study design;
- and unavailable full-text articles.

### 3.2. Characteristics of Included Studies

The principal characteristics of the included studies are summarized in Table 2. Most included investigations were observational, cross-sectional, or screening-based cohort studies evaluating HPV genotype distribution, cervical cancer screening outcomes, molecular biomarkers, vaccination-related public health data, or vaginal microbiome composition in Eastern European populations.

The included studies originated primarily from Romania, Croatia, Latvia, and multicenter Eastern European or Eastern Europe–Central Asia cohorts. Study populations included women participating in organized or opportunistic cervical cancer screening programs, women with abnormal cervical cytology, persistent high-risk HPV infection, or histologically confirmed cervical intraepithelial neoplasia (CIN).

Several studies evaluated HPV genotype prevalence and genotype diversity within screening populations. Kaliterna et al. [1] investigated genotype-specific high-risk HPV distribution among HPV-positive women from Croatia and reported HPV16 and HPV31 as the predominant genotypes. Stasulane et al. [4] analyzed HPV genotype prevalence in women participating in the Latvian national cervical cancer screening program and identified HPV16, HPV68, and HPV31 among the most prevalent high-risk HPV genotypes.

Multiple Romanian studies focused on molecular biomarker evaluation and cervical lesion risk stratification. Camarasan et al. [13] evaluated the role of CINtec PLUS cytology in women with abnormal cervical cytology and reported improved specificity for identification of CIN2+ lesions. Brăila et al. [15] investigated the relationship between cervicovaginal infections, persistent high-risk HPV infection, and cervical intraepithelial neoplasia progression in Romanian women, supporting the potential role of vaginal dysbiosis in cervical carcinogenesis, while Munteanu et al. [16] evaluated clinical and virological characteristics associated with CINtec PLUS positivity in Romanian CIN patients.

Regional public health analyses also addressed disparities in HPV vaccination implementation and cervical cancer screening infrastructure across Eastern Europe and Central Asia. Ussai et al. [17] reported substantial heterogeneity regarding vaccination uptake, screening accessibility, and implementation of HPV-based screening strategies throughout the region.

Overall, the included studies demonstrated considerable heterogeneity regarding study populations, screening settings, laboratory methodologies, and evaluated clinical outcomes.

### 3.3. HPV Prevalence and Genotype Distribution

HPV prevalence across the included Eastern European screening populations ranged from approximately 12% to over 20% [4,8]. Higher prevalence rates were generally reported in women with abnormal cytology, persistent high-risk HPV infection, or referral-based populations compared with organized screening cohorts.

HPV16 was consistently identified as the predominant oncogenic genotype across all included studies [1,4,8,9]. However, several investigations also demonstrated substantial prevalence of non-16/18 high-risk HPV genotypes, particularly HPV31, HPV51, HPV52, HPV66, and HPV68 (Table 3) [1,4,8,15]. In certain cohorts, non-16/18 high-risk HPV genotypes represented a substantial proportion of detected infections. Kaliterna et al. reported HPV31 in 22.4%, HPV52 in 11.0%, and HPV68 in 9.3% of HPV-positive Croatian women, whereas Stasulane et al. identified HPV68, HPV31, and HPV52 among the most prevalent genotypes in Latvian screening populations [1,4].

Romanian studies identified HPV16, HPV31, and HPV52 among the most clinically relevant genotypes associated with CIN2/CIN3 lesions [9,15,16]. Croatian and Latvian cohorts demonstrated increased prevalence of HPV31 and HPV68 in HPV-positive women [1,4].

Multiple HPV infections were frequently reported, particularly among younger women and women with persistent high-risk HPV infection. Several studies identified associations between multiple high-risk HPV infections and abnormal cytological findings, biomarker positivity, or increased lesion severity [1,17].

### 3.4. Molecular Biomarkers and Screening Implications

Several included studies evaluated molecular biomarkers as complementary approaches for cervical cancer screening and risk stratification in high-risk HPV-positive women (Table 4).

CINtec PLUS cytology, based on simultaneous p16 and Ki-67 expression, demonstrated increased specificity for identifying clinically significant cervical lesions compared with HPV DNA testing alone [9,12,13]. Positive p16/Ki-67 dual staining was consistently associated with CIN2+ lesions, persistent high-risk HPV infection, and abnormal cytological findings [13,18].

Camarasan et al. [9] reported improved specificity of CINtec PLUS cytology for identifying high-grade lesions in Romanian women with abnormal cytology. Similarly, Munteanu et al. [16] demonstrated associations between CINtec positivity and CIN2/CIN3 lesions in high-risk HPV-positive patients.

HPV E6/E7 mRNA testing was also evaluated as a molecular triage approach for identifying transcriptionally active high-risk HPV infections [12,14]. Studies reported stronger associations between HPV mRNA positivity and high-grade lesions compared with HPV DNA positivity alone [12]. Several investigations suggested that HPV E6/E7 mRNA testing may improve risk stratification among women with persistent high-risk HPV infection.

Extended HPV genotyping was additionally reported as a potentially useful strategy for identifying women with non-16/18 high-risk HPV infections associated with increased risk of cervical neoplasia [10,11].

### 3.5. HPV Vaccination and Public Health Implications

Several studies evaluated HPV vaccination implementation and cervical cancer prevention strategies across Eastern Europe [6,19]. Considerable heterogeneity in vaccination coverage and national immunization policies was reported among countries within the region (Table 5) [9,16].

The 9-valent HPV vaccine was identified as particularly relevant for Eastern European populations due to its coverage of several frequently reported high-risk HPV genotypes, including HPV31, HPV52, and HPV58 [6,20]. Nevertheless, studies consistently reported lower vaccination uptake in several Eastern European countries compared with Western European populations [20,21].

Barriers to vaccine implementation included socioeconomic disparities, limited public awareness, healthcare accessibility limitations, and vaccine hesitancy [6,20]. Several studies also highlighted persistent disparities regarding organized screening participation and access to HPV-based molecular diagnostics across the region [20].

### 3.6. Vaginal Microbiome and HPV Persistence

Several included studies investigated associations between vaginal microbiome composition, persistent high-risk HPV infection, and cervical lesion severity [15,16,22].

*Lactobacillus*-dominant vaginal microbiota were consistently associated with cervical health, lower inflammatory status, and increased likelihood of HPV clearance [16,17]. In particular, *Lactobacillus crispatus*-dominant communities were associated with lower prevalence of persistent high-risk HPV infection and greater microbiome stability [17].

Conversely, vaginal dysbiosis characterized by increased microbial diversity and predominance of anaerobic bacterial species was associated with persistent high-risk HPV infection and higher-grade cervical lesions [15,18]. *Gardnerella vaginalis*, *Prevotella* species, *Atopobium vaginae*, and *Sneathia* species were among the microorganisms most frequently associated with dysbiotic microbiota in high-risk HPV-positive women [17,18,22].

Several studies also reported associations between dysbiotic microbiota, increased inflammatory cytokine expression, and molecular biomarker positivity, including CINtec PLUS dual staining and HPV E6/E7 mRNA positivity (Table 6) [18].

### 3.7. Screening Modalities and Clinical Implications

Included studies demonstrated substantial heterogeneity regarding cervical cancer screening implementation throughout Eastern Europe [20,23]. Conventional cytology-based screening remained widely used in several countries, whereas HPV DNA-based screening approaches were variably implemented (Table 7).

HPV DNA testing demonstrated higher sensitivity for identifying women at risk for CIN2+ lesions compared with cytology alone [11,12]. However, several studies also highlighted lower specificity associated with HPV DNA testing, particularly in younger populations with transient infections [11].

Molecular triage approaches, including CINtec PLUS cytology and HPV E6/E7 mRNA testing, were reported to improve specificity and reduce unnecessary colposcopy referrals among high-risk HPV-positive women [13,15,21].

Extended HPV genotyping was additionally reported as a potentially valuable strategy in Eastern European populations characterized by broad genotype heterogeneity and frequent non-16/18 high-risk HPV infections [10,11,12].

### 3.8. Risk of Bias Assessment

Overall, most included studies demonstrated moderate-to-high methodological quality, with NOS scores ranging from 6 to 8. The most common methodological concerns were selection bias related to single-center or referral-based populations, limited longitudinal follow-up, and heterogeneity in HPV detection methods, biomarker interpretation, and microbiome assessment protocols. Although the overall risk of bias was considered low to moderate, these factors should be considered when interpreting the findings.

## 4. Discussion

This section integrates findings from the studies included in the qualitative synthesis together with selected external literature used to contextualize the findings within current international evidence and screening recommendations.

This systematic review provides a comprehensive synthesis of recent evidence regarding HPV genotype distribution, molecular biomarkers, vaginal microbiome composition, and cervical cancer prevention strategies in Eastern European populations. The findings demonstrate substantial regional heterogeneity in HPV genotype prevalence, screening implementation, vaccination coverage, and access to molecular diagnostic technologies throughout the region [1,4,9,15]. Although HPV16 remained the predominant oncogenic genotype across all included studies, several investigations consistently identified non-16/18 high-risk HPV genotypes, particularly HPV31, HPV51, HPV52, HPV66, and HPV68, as clinically relevant contributors to persistent infection and high-grade cervical lesions [1,11,15,16].

One of the principal findings of this review was the broad genotype diversity observed across Eastern European populations. While HPV16 continues to demonstrate the strongest association with persistent infection and cervical carcinogenesis, multiple studies reported high prevalence of additional oncogenic genotypes that approached or exceeded the prevalence of HPV18 in certain cohorts [1,7,10]. These findings suggest that the epidemiological profile of HPV infection in Eastern Europe may differ from that traditionally described in Western European populations, where HPV16 and HPV18 have historically dominated screening and vaccination strategies.

The predominance of non-16/18 high-risk HPV genotypes has important implications for cervical cancer screening algorithms and molecular triage approaches [8,11]. Current screening recommendations in many settings continue to prioritize HPV16 and HPV18 for immediate colposcopy referral and risk stratification [11,12]. However, the findings of this review suggest that screening algorithms focused predominantly on HPV16 and HPV18 may inadequately capture oncogenic risk in Eastern European populations characterized by broader genotype heterogeneity. Consequently, extended HPV genotyping may represent an important strategy for improving individualized risk assessment and identifying women at increased risk for persistent infection and progression to CIN2/CIN3 lesions.

Another important finding of this review was the frequent identification of multiple HPV infections, particularly among younger women and women with persistent high-risk HPV infection [1,4,9,15]. Several included studies reported associations between multiple oncogenic HPV infections, abnormal cytological findings, and increased biomarker positivity [1,4,15,19]. Although the precise clinical significance of multiple high-risk HPV infections remains incompletely understood, previous investigations have suggested that co-infections may contribute to chronic inflammatory responses, prolonged viral persistence, and increased risk of cervical epithelial transformation [4,7,9].

This review also highlights the ongoing transition from conventional cytology-based cervical cancer screening toward molecular risk-based screening approaches. Although cytology remains widely used in several Eastern European countries, multiple studies demonstrated the superior sensitivity of HPV DNA testing for identifying women at risk for CIN2+ lesions [11,14,15]. Nevertheless, HPV DNA testing alone demonstrated lower specificity, particularly in younger women with transient infections [10,11]. As a result, molecular triage strategies capable of improving specificity while reducing unnecessary colposcopy referrals have become increasingly important [15].

Among the evaluated molecular biomarkers, CINtec PLUS cytology emerged as one of the most clinically relevant adjunctive screening tools. Multiple included studies demonstrated strong associations between p16/Ki-67 dual staining positivity and CIN2/CIN3 lesions [13,18,19,21]. Simultaneous expression of p16 and Ki-67 within the same cervical epithelial cell reflects deregulated cell-cycle activity associated with transforming HPV infection and therefore represents a biologically meaningful marker of oncogenic progression [18,19,20]. Several investigations suggested that CINtec PLUS cytology may improve triage of high-risk HPV-positive women and reduce unnecessary invasive procedures in populations characterized by high-risk HPV prevalence [19].

Similarly, HPV E6/E7 mRNA testing demonstrated promising clinical utility for identifying transcriptionally active high-risk HPV infections associated with increased malignant potential [12,14]. Unlike HPV DNA testing, which detects viral genetic material regardless of biological activity, HPV mRNA assays evaluate expression of the viral oncogenes directly involved in carcinogenesis [20,21]. Several studies included in this review reported stronger associations between HPV E6/E7 mRNA positivity and high-grade cervical lesions compared with HPV DNA positivity alone [23]. These findings support the potential integration of HPV mRNA testing into future risk-based screening algorithms, particularly among women with persistent high-risk HPV infection or equivocal cytological findings [9,10,12].

Another important aspect highlighted by this review was the emerging role of the vaginal microbiome in HPV persistence and cervical carcinogenesis [1,14]. *Lactobacillus*-dominant microbiota, particularly *Lactobacillus crispatus*-dominant communities, were consistently associated with cervical health, lower inflammatory status, and increased likelihood of viral clearance [16,17,22]. Conversely, dysbiotic vaginal microbiota characterized by increased microbial diversity and predominance of anaerobic bacterial species were consistently associated with persistent high-risk HPV infection and progression to high-grade cervical lesions across several included studies [1,14,16].

Several studies identified *Gardnerella vaginalis*, *Prevotella* species, *Atopobium vaginae*, and *Sneathia* spp. as microorganisms frequently associated with dysbiotic vaginal microbiota in high-risk HPV-positive women [15,17,18]. Although causal relationships remain incompletely understood, emerging evidence suggests that dysbiotic vaginal microbiota may contribute to epithelial barrier disruption, altered mucosal immunity, chronic inflammatory responses, and increased oncogenic viral activity associated with persistent high-risk HPV infection [1,14]. Increased concentrations of inflammatory cytokines, including interleukin-6 (IL-6), interleukin-1β (IL-1β), and tumor necrosis factor-alpha (TNF-α), have also been reported in women with persistent high-risk HPV infection and vaginal microbiome instability [14,16,22].

Importantly, several investigations also reported associations between vaginal dysbiosis and increased positivity rates for molecular biomarkers such as CINtec PLUS cytology and HPV E6/E7 mRNA testing [17,18]. These findings suggest that microbiome-related alterations may influence not only viral persistence but also transforming HPV infection and progression toward cervical neoplasia. Nevertheless, microbiome-related evidence remains relatively limited in Eastern European populations, and substantial methodological heterogeneity persists regarding sequencing platforms, microbiome classification systems, and bioinformatic analysis pipelines.

The findings of this review also have important implications for HPV vaccination strategies in Eastern Europe. Although the 9-valent HPV vaccine provides coverage against several of the most prevalent non-16/18 high-risk HPV genotypes identified in this review, vaccination uptake remains inconsistent across many countries within the region [6,15,16,20]. Socioeconomic disparities, healthcare infrastructure limitations, vaccine hesitancy, and reduced participation in organized screening programs continue to contribute to the persistent burden of HPV-related disease in Eastern Europe [15,16].

In addition, substantial disparities remain regarding implementation of organized HPV-based screening programs and access to molecular diagnostic technologies. Several countries continue to rely predominantly on opportunistic cytology-based screening approaches despite growing evidence supporting HPV-based primary screening and molecular risk stratification [9,15,16]. Strengthening national screening infrastructure and improving accessibility of molecular diagnostic technologies therefore remain important public health priorities throughout the region.

The findings support the growing importance of extended HPV genotyping, molecular biomarker integration, and microbiome-related approaches in future precision-based cervical cancer screening strategies.

Future prospective multicenter studies are needed to evaluate the combined predictive value of HPV genotyping, molecular biomarkers, and vaginal microbiome profiling within organized screening programs. In addition, improving vaccination coverage, strengthening organized HPV-based screening programs, and expanding access to molecular diagnostic technologies remain essential priorities for reducing the burden of cervical cancer throughout Eastern Europe.

### Limitations

Several limitations should be considered when interpreting the findings of this systematic review. Despite a comprehensive search strategy, only seven studies met the predefined eligibility criteria and were included in the final qualitative synthesis. Moreover, the available evidence originated predominantly from Romania, Croatia, and Latvia, together with one regional public health analysis. This limited geographical representation may not fully capture the diversity of HPV epidemiology, molecular biomarker utilization, vaginal microbiome characteristics, screening practices, and vaccination implementation across all Eastern European countries. Therefore, the conclusions should be interpreted as reflective of the currently available evidence rather than representative of the entire region. Furthermore, this limited representation reflects the current scarcity of published HPV-related research from several Eastern European countries rather than an absence of disease burden within the region.

Although the search strategy incorporated both the regional term “Eastern Europe” and country-specific keywords to maximize retrieval sensitivity, relevant studies from some countries formally classified as Eastern Europe may not have been identified. Variations in database indexing practices, publication language, and research output among countries may have contributed to the underrepresentation of certain populations, potentially leading to an underestimation of true regional variability. In addition, differences in national screening program implementation, healthcare accessibility, and HPV vaccination coverage may have influenced reported outcomes and contributed to observed inter-country variability.

The interpretation of findings was further limited by substantial methodological heterogeneity among the included studies. Differences in study design, study populations, sample size, study periods, screening settings, HPV detection and genotyping assays, molecular biomarker assessment methods, microbiome characterization techniques, and the availability of vaccination-related data reduced comparability across studies and precluded quantitative meta-analysis. Consequently, a qualitative narrative synthesis was considered the most appropriate approach.

Most included studies were observational and cross-sectional in design, limiting the ability to establish causal relationships between HPV genotype distribution, vaginal microbiome alterations, molecular biomarker expression, and progression to cervical neoplasia. Furthermore, evidence regarding vaginal microbiome composition and HPV E6/E7 mRNA testing in Eastern European populations remains relatively limited, restricting the strength of conclusions that can currently be drawn regarding their clinical utility and predictive value.

Finally, several references discussed in the Section 4 were not included in the final qualitative synthesis but were incorporated to contextualize the findings within the broader international literature and current screening recommendations. These references were used solely to support interpretation of the results and should be distinguished from the evidence generated by the studies formally included in this review.

Despite these limitations, this review provides an updated synthesis of current evidence regarding HPV genotype diversity, molecular biomarkers, vaginal microbiome composition, and cervical cancer prevention strategies in Eastern Europe. The findings highlight important knowledge gaps and support the need for larger multicenter studies involving a broader range of Eastern European countries to strengthen the evidence base and inform future cervical cancer prevention policies in the region.

## 5. Conclusions

This systematic review highlights the evolving landscape of HPV-related cervical cancer prevention in selected Eastern European populations represented in the available literature, particularly Romania, Croatia, and Latvia, and emphasizes the growing importance of integrated molecular and microbiome-based approaches in contemporary screening strategies. Although HPV16 remained the predominant oncogenic genotype across the included studies, substantial prevalence of non-16/18 high-risk HPV genotypes, particularly HPV31, HPV51, HPV52, HPV66, and HPV68, demonstrated considerable genotype heterogeneity throughout Eastern European populations.

The findings of this review suggest that cervical cancer screening algorithms focused primarily on HPV16 and HPV18 may not fully capture oncogenic risk in populations characterized by broader genotype diversity. Consequently, extended HPV genotyping may represent an important component of future risk-based cervical cancer screening strategies in Eastern Europe.

Molecular biomarkers, including CINtec PLUS cytology and HPV E6/E7 mRNA testing, demonstrated important clinical utility for improving specificity in the identification of clinically significant cervical lesions among high-risk HPV-positive women. These approaches may contribute to more accurate risk stratification and reduction in unnecessary colposcopy referrals within organized screening programs.

This review also highlights the emerging association between vaginal microbiome composition, persistent high-risk HPV infection, and cervical carcinogenesis. *Lactobacillus*-dominant microbiota were consistently associated with cervical health and viral clearance, whereas dysbiotic vaginal microbiota characterized by increased microbial diversity and anaerobic bacterial predominance were associated with persistent high-risk HPV infection and progression to high-grade cervical lesions. Although microbiome-related evidence remains limited, current findings suggest that microbiome profiling may contribute to future precision-based cervical cancer prevention strategies.

Substantial disparities in HPV vaccination uptake, screening accessibility, and implementation of molecular diagnostic technologies continue to persist across Eastern Europe. Despite the broad theoretical protection provided by the 9-valent HPV vaccine against several prevalent non-16/18 high-risk HPV genotypes identified in this review, vaccination coverage remains inconsistent throughout the region.

Collectively, the findings of this systematic review support the growing importance of integrating extended HPV genotyping, molecular biomarkers, HPV mRNA testing, and microbiome-related approaches into future cervical cancer prevention programs in Eastern Europe. Future prospective multicenter studies are needed to further evaluate the combined predictive value of molecular biomarkers and vaginal microbiome profiling within organized HPV-based screening programs. Strengthening organized screening infrastructure, expanding vaccination coverage, and improving access to molecular diagnostic technologies remain essential priorities for reducing the burden of cervical cancer throughout the region.

## Figures and Tables

**Table 1:** Methodological Quality Assessment and Risk of Bias of Included Studies.

Study	Study Design	Main Limitation	NOS Score	Overall Quality	Principal Risk of Bias
Kaliterna et al. (2025) [1]	Cross-sectional	Single-country cohort	7/9	High	Selection bias due to recruitment from a single national population
Stasulane et al. (2025) [4]	Screening cohort	Limited longitudinal follow-up	8/9	High	Potential attrition bias and inability to assess long-term outcomes
Berza et al. (2024) [8]	Cross-sectional	Cross-sectional design and lack of follow-up	7/9	High	Selection bias and inability to infer causal relationships
Camarasan et al. (2024) [9]	Observational	Moderate sample size	6/9	Moderate	Referral population bias and limited statistical power
Brăila et al. (2025) [15]	Observational	Single-center cohort	7/9	High	Selection bias associated with single-center recruitment
Munteanu et al. (2025) [16]	Observational/Modeling	Single-center population	7/9	High	Selection bias and limited external validity
Ussai et al. (2025) [17]	Regional public health analysis	Heterogeneous national datasets	7/9	High	Reporting heterogeneity and variability in national surveillance systems

**Table 2:** Characteristics of Included Studies.

Author (Year)	Country	Study Design	Study Population	Sample Size	Main Focus	Main Findings
Kaliterna et al. (2025) [1]	Croatia	Cross-sectional	HPV-positive women	294	HPV genotype distribution and microbiome	HPV16 and HPV31 predominant; frequent multiple infections
Stasulane et al. (2025) [4]	Latvia	Screening cohort	National screening population	4000+	HPV prevalence and genotype distribution	HPV16, HPV68, and HPV31 frequently identified
Berza et al. (2024) [8]	Latvia	Cross-sectional	Women participating in screening	2000+	High-risk HPV prevalence and associated factors	High prevalence of non-16/18 high-risk HPV genotypes
Camarasan et al. (2024) [9]	Romania	Observational	Women with abnormal cytology	98	CINtec PLUS cytology	Improved specificity for CIN2+ lesions
Brăila et al. (2025) [15]	Romania	Observational	Women with HPV infection and cervical intraepithelial neoplasia	94	Cervicovaginal infections and HPV-related CIN progression	Persistent high-risk HPV infection and cervicovaginal dysbiosis were associated with cervical lesion progression
Munteanu et al. (2025) [16]	Romania	Observational/modeling	CIN patients	359	CINtec PLUS positivity and risk prediction	Significant association with CIN2/CIN3 lesions
Ussai et al. (2025) [17]	Eastern Europe/Central Asia	Public health analysis	Regional populations	Multinational	HPV vaccination implementation	Marked disparities in vaccine uptake and screening

**Table 3:** Most Frequently Reported High-Risk HPV Genotypes in Eastern Europe.

Country	Most Frequent High-Risk HPV Genotypes	Notable Findings
Romania	HPV16, HPV31, HPV52	Strong association with CIN2/CIN3 lesions
Croatia	HPV16, HPV31, HPV51	High genotype diversity and multiple infections
Latvia	HPV16, HPV68, HPV31	Increased prevalence of non-16/18 genotypes

**Table 4:** Molecular Biomarkers Evaluated in Included Studies.

Biomarker	Clinical Role	Main Findings	Clinical Implications
CINtec PLUS (p16/Ki-67)	Triage of high-risk HPV-positive women	Increased specificity for CIN2+ lesions	Reduced unnecessary colposcopy referrals
HPV E6/E7 mRNA	Detection of transforming infection	Strong association with high-grade lesions	Improved risk stratification
Extended HPV genotyping	Identification of non-16/18 high-risk HPV infections	Better characterization of genotype diversity	Improved individualized screening
HPV DNA testing	Primary screening method	High sensitivity for CIN2+ detection	Effective for organized screening programs

**Table 5:** Implications of HPV Genotype Distribution for Vaccination Strategies.

Vaccine Type	Covered Genotypes	Relevance in Eastern Europe
Bivalent vaccine	HPV16, HPV18	Partial regional genotype coverage
Quadrivalent vaccine	HPV6, HPV11, HPV16, HPV18	Protection against genital warts and major oncogenic genotypes
9-valent vaccine	HPV6, HPV11, HPV16, HPV18, HPV31, HPV33, HPV45, HPV52, HPV58	Broadest protection for circulating high-risk HPV genotypes

**Table 6:** Vaginal Microbiome Findings in HPV-Positive Women.

Microbiome Pattern	Associated Clinical Findings
*Lactobacillus*-dominant microbiota	HPV clearance and cervical health
*Lactobacillus crispatus* predominance	Lower persistence of high-risk HPV infection
Increased microbial diversity	Persistent high-risk HPV infection
Anaerobic dysbiosis	Higher risk of CIN2/CIN3 lesions
*Gardnerella*/*Prevotella* predominance	Increased inflammatory response
Microbiome instability	Increased biomarker positivity

**Table 7:** Screening Modalities and Clinical Implications.

Screening Method	Advantages	Limitations	Clinical Implications
Cytology (Pap smear)	Widely available and low cost	Lower sensitivity and interobserver variability	Requires repeated screening
HPV DNA testing	High sensitivity for CIN2+ detection	Lower specificity	Effective primary screening tool
Extended HPV genotyping	Improved risk stratification	Increased laboratory costs	Better identification of non-16/18 high-risk HPV infections
CINtec PLUS cytology	Increased specificity	Limited availability	Reduced unnecessary colposcopy referrals
HPV E6/E7 mRNA testing	Detection of transforming infection	Higher cost and limited accessibility	Better prediction of lesion progression

## Data Availability

The data presented in this study are available on request from the corresponding author due to legal reasons.
